# Evaluation of the criteria for angiotomography indications in the diagnosis of carotid and vertebral arterial injury associated with blunt trauma

**DOI:** 10.1186/1749-7922-5-17

**Published:** 2010-06-26

**Authors:** Goulart Gladstone, Porta Maria Pereira Rina, Poggetti Sérgio Renato, Fontes Belchor, Júnior Lourenço de Souza Almerindo, Gattas Gabriel, Birolini Dario

**Affiliations:** 1 Hospital das Clínicas da Faculdade de Medicina da Universidade de São Paulo (HCFMUSP), Av. Dr. Enéas de Carvalho Aguiar, 255 8° and. s/8131, São Paulo/SP, CEP 05403-000, Brazil; 2 Departamento de Cirurgia Vascular do HCFMUSP, Brazil; 3 Faculdade de Medicina da Universidade de São Paulo (FMUSP), Brazil; 4 Departamento de Cirurgia de Emergência do HCFMUSP, Brazil; 5 Departamento de Radiologia do HCFMUSP, Brazil

## Abstract

**Background:**

Blunt carotid and vertebral artery injury (BCVI) occur infrequently. The incidence of this type of injury is difficult to determine as many emergency room patients are neurologically asymptomatic. The statistics have not been reported in Brazil. The objectives of the current study were: To evaluate the accuracy of criteria used to recommend angiotomography in the diagnosis of cervical BCVI in 100 patients with blunt cervical trauma in the trauma services section of a Brazilian quaternary care hospital.

**Methods:**

During a 30-month (2006-2008), all patients admitted to the emergency room of Hospital das Clínicas da Faculdade de Medicina da Universidade de São Paulo with blunt cervical trauma and potential risk of cervical vessel injury, were subjected to cervical angiotomography to diagnose BCVI. The data analyzed are presented as mean ± standard deviation, and statistical analyses included Chi-square and Fisher's exact tests, and the Mann-Whitney test.

**Results:**

During the study period 2467 blunt trauma patients were admitted. In 100 patients that met the criteria for inclusion in the study, angiotomography identified 23 with BCVI, including 17 males and six females. The mean patient age was 34.81 ± 14.84 years. Car crash (49%) and car-pedestrian accidents (24%) were the most frequent causes of injury. Ten patients had internal carotid artery injuries, two patients had common carotid artery injuries, and 11 patients had vertebral artery injuries. Seven patients presented with Degree I arterial injuries, 10 patients presented with Degree II artery injuries, four patients presented with Degree IV artery injuries, one patient presented with a Degree V artery injury, and one patient had a carotid fistula. Seven out of the 23 patients with BCVI (30.4%) presented with cervical vertebrae fractures, and 11 out of the 23 patients with BCVI (47.8%) presented with facial fractures (LeFort II and III).

**Conclusions:**

Although there is no consensus regarding the criteria that should be used to indicate angiotomography for BCVI diagnosis, we conclude that the criteria used in the current study led to a diagnosis of BCVI in 0.93% of 2,467 trauma patients, BCVI injuries were associated with more severe traumas and did not affect mortality.

## Introduction

Blunt carotid and vertebral artery injury (BCVI) is infrequent, but may have serious repercussions. The incidence of this type of injury is difficult to evaluate as many emergency room patients are neurologically asymptomatic or have symptoms attributed to cranial trauma or to other associated injuries. Previous studies estimated that BCVI injuries remain undiagnosed in two-thirds of patients [1,2]. More recent statistics show an incidence of BCVI lesions in 0.24% to 0.33% of trauma patients with some symptoms of neurological impairment [3,4]. Therefore, the high index of suspicion is fundamental to the diagnosis of these lesions in blunt cervical trauma. To our knowledge, this is the first study to examine the incidence of BCVI in Brazil. Given the low incidence of these traumas, their actual morbidity and mortality have not been clearly established in the literature. Of particular importance, many BCVIs are not diagnosed or treated before an ischemic cerebral event occurs [5]. In the 1974'-*s, studies identified that the most common pathophysiologic mechanism is an intimal tear with subsequent thrombosis. While the symptoms are generally those of carotid insufficiency, a diagnosis of cervical carotid trauma is seldom made clinically because the entity is confused with intracranial injury [2,6].

Several laboratory tests and imaging studies are frequently required in the emergency room for the evaluation of trauma. However, imaging exams to identify cervical vessel lesions are not performed routinely during initial trauma care. Angiography is considered the 'gold standard' exam for the identification of vascular lesions. The duplex scan has 86% sensitivity, but is limited in its ability to identify carotid artery lesions near the base of the skull. Angiotomography is sensitive enough to identify general anatomical lesions, and it could also be useful for identifying vascular lesions. During initial trauma assessment, computerized tomography is a common diagnostic method [1,2,5,7,8]. Magnetic resonance angiography has the ability to produce images of the neck and head simultaneously and to detect early cerebral infarction without the use of contrast [1,5,8,9].

In the 1990's, studies using angiography as a diagnostic method in populations at risk for BCVI demonstrated that these lesions are rare, corresponding to 1% of all blunt traumas admitted to hospital. Due to limited experience with BCVI in trauma centers, standardized diagnostic and therapeutic approaches to these injuries have not been established. Furthermore, the current approach to BCVI classification has not been unanimously accepted. These limitations have restricted the development of a practical, safe, and universal approach to handling BCVI cases [5]. Although BCVI treatment approaches are debated, all current modalities of treatment, whether clinical or endovascular, depend on the clinical situation, the experience of the medical team, and, above all, the exact characterization of the location and severity of the lesion using an appropriate diagnostic method.

### Objective

To evaluate the accuracy of criteria used to recommend angiotomography in the diagnosis of cervical BCVI in 100 patients with blunt cervical trauma in the trauma services section of a Brazilian quaternary care hospital.

## Materials and methods

The current study was approved by the Ethics Committee for Analysis of Research Projects - CAPPesq of the Hospital das Clínicas da Faculdade de Medicina da Universidade de São Paulo. It is based on data obtained from medical records of patients who suffered blunt trauma and were admitted to the Emergency Department of the Hospital das Clínicas da Faculdade de Medicina da Universidade de São Paulo (HCFMUSP) from July 2006 to December 2008 using clinical and/or radiographic data that indicated a potential risk of BCVI.

Inclusion criteria in the current study were designed based on eleven previously published criteria. The following inclusion criteria were used for cervical angiotomography: 1) unilateral neurological deficit not justified by CT of the skull; 2) cerebral infarction identified in the CT of the skull; 3) nonexpanding cervical hematoma; 4) epistaxis; 5) anisocoria/signs of Horner syndrome; 6) score lower than eight on the Glasgow coma scale without supporting findings on the CT of the skull; 7) cervical spine fracture; 8) fracture of the base of the skull; 9) fracture of facial bones with a LeFort II and/or III classification; 10) signs of a seatbelt positioned above the clavicle; and 11) cervical tremor or blow.

The patients underwent cervical angiotomography if they were hemodynamically normal. All angiotomographies were performed using a GE, Light Speed Ultra, multi-slice helical CT Scanner with 8 slices per rotation.

The following BCVI alterations were classified according to degrees of severity from one to five: 1) Grade I, luminal irregularities of the artery or dissections with stenosis comprising less than 25% of the lumen; 2) Grade II, dissections or intramural hematomas with stenosis greater than or equal to 25% of the lumen, the intraluminal thrombus, or the raised patches in the intima; 3) Grade III, pseudoaneurysm; 4) Grade IV, occlusions; and 5) Grade V, sections with hemorrhaging. Fistulas were classified separately.

Age, sex, trauma mechanisms, and vital signs were obtained during the initial treatment of the trauma patient, and the respiratory rate (RR), heart rate (HR), arterial O_2 _saturation, arterial pressure (AP), and Glasgow coma scale score were analyzed. The revised trauma score (RTS) and injury severity score (ISS) of the lesion were determined, and the probability of survival based on the trauma injury severity score (TRISS) was calculated based on the correlation between the RTS, the ISS of the lesion, the trauma mechanism, and the age of the patient.

All of these indices were calculated in the patient populations without BCVI (Group I) and with BCVI (Group II). The data is presented as means and standard deviations of the means, and the statistical analyses were performed using Chi-Squared and Fisher's Exact tests, and the Mann-Whitney test; p-values ≤ 0.05 were considered statistically significant.

## Results

In the 30-month period of the current study, which took place from July 2006 to December 2008, a total of 2,467 blunt trauma patients were admitted to the Emergency Surgery Service of the III Division of Clinical Surgery of Hospital das Clínicas da Faculdade de Medicina da Universidade de São Paulo. Out the 2,467 blunt trauma patients, 100 presented criteria for inclusion in the study and underwent cervical angiotomography. Out of these 100 patients, 61 were scanned immediately after clinical evaluation in the emergency room and 39 were scanned after hemodynamic stabilization.

The time that elapsed between admission and the cervical angiotomography procedure, which varied from one to 18 days, was analyzed and no statistically significant difference was found between Groups I and II (Table 1). In the group of 100 patients, angiotomography identified 77 patients without BCVI (Group I) and 23 patients with BCVI (Group II). The incidence of BCVI represented 0.93% of the total of the patients diagnosed with blunt trauma during the 30-month period. The average age of the total population of 100 patients was 34.81 years with a standard deviation of 14.84 years and a variation of 7 to 77 years. In the group of 77 patients without BCVI (Group I), the average age was 35.43 ± 15.49 years; in the group of 23 patients with BCVI (Group II), the average age was 32.74 ± 12.51 years.

**Table 1:** Time between admission and cervical angiotomography according to whether BCVI were absent (Group I) or present (Group II) in the 100 patients selected for cranial angiotomography.

Time	Group		Total	p-value
	I (without Injury)	II (with injury)		
**Immediate**	49 (63.6%)	12 (52.2%)	61 (61%)	0.3227
**Not immediate**	28 (36.4%)	11 (47.8%)	39 (39%)	
**Total**	77	23	100	

Of the total population of 100 patients, 85 (85%) were male and 15 (15%) were female. Of the 85 male patients, 68 did not present with BCVI (Group I), and 17 did present with BCVI (Group II). Of the 15 female patients, nine did not present with BCVI (Group I) and six did present with BCVI (Group II). There was no statistically significant difference between Groups I and II with regard to sex or age.

The mechanisms of trauma for the total population of 100 patients included: motor vehicle collisions (49 patients); car-pedestrian accidents (24 patients); aggression (4 patients); falls from heights (18 patients); and other mechanisms (5 patients). In the group of 77 patients without BCVI (Group I), the distribution of trauma mechanisms was: motor vehicle collisions (36 patients); car-pedestrian accidents (20 patients); aggression (4 patients); falling from heights (14 patients); and other mechanisms (3 patients). In the group of 23 patients with BCVI (Group II), the distribution of trauma mechanisms was: motor vehicle collisions (13 patients); car-pedestrian accidents (4 patients); aggression (no patients); falling from heights (4 patients); and other mechanisms (2 patients). There was no statistically significant difference between Groups I and II with regard to the mechanisms of trauma.

Vital sign values for the total population of 100 patients collected during the initial assessment in the emergency room were: systolic blood pressure (SBP) of 123.09 ± 22.93 mm Hg, diastolic blood pressure (DBP) of 77.91 ± 19.94 mm Hg, respiratory rate (RR) of 15.82 ± 11.05 irpm, heart rate (HR) of 98.91 ± 21.87 bpm, and arterial saturation of O_2 _of 93.23 ± 7.94%. Patients without BCVI (Group I) had an average SPB of 123.35 ± 23.61 mm Hg, and patients with BCVI (Group II) had an average SPB of 122.22 ± 20.96 mm Hg. Patients in Group 1 had an average DBP of 79.16 ± 18.29 mm Hg, and patients in Group II had an average DPB of 73.74 ± 24.69 mm Hg. Patients in Group I had an average RR of 16.35 ± 12.88 irpm, and patients in Group II an average RR of 14.04 ± 3.96 irpm. Patients in Group I had an average HR of 97.92 ± 20.13 bpm, and patients in Group II had an average HR of 102.22 ± 27.17 bpm. Patients in Group I had an average arterial saturation of O_2 _of 93.08 ± 8.17 mm Hg, and patients in Group II had an average arterial saturation of O_2 _of 93.74 ± 7.28 mm Hg. There was no statistically significant difference between Groups I and II with regard to SBP, DBP, RR, HR, or arterial saturation of O_2 _(Table 2).

**Table 2:** Vital signs in 100 patients that underwent cervical angiotomography.

	Groups		Total	p-value
	I (without Injury)	II (with injury)		
**SBP (mm Hg)**				
Average ± SD	123.35 ± 23.61	122.22 ± 20.96	123.09 ± 22.93	0.6830
Median	127	120	127	
Minimum - Maximum	60 - 165	85 - 160	60 - 165	
**DBP (mm Hg)**				
Average ± SD	79.16 ± 18.29	73.74 ± 24.69	77.91 ± 19.94	0.1851
Median	80	70	80	
Minimum - Maximum	30 - 120	19.13 - 130	19.13 - 130	
**RR (irpm)**				
Average ± SD	16.35 ± 12.38	14.04 ± 3.96	15.82 ± 11.05	0.9606
Median	14	15	15	
Minimum - Maximum	0 - 115	5 - 20	0 - 115	
**HR (bpm)**				
Average ± SD	97.92 ± 20.13	102.22 ± 27.17	98.91 ± 21.87	0.2125
Median	95	100	96	
Minimum - Maximum	45 - 145	14 - 150	14 - 150	
**Arterial Saturation of O_2 _(%)**				
Average ± SD	93.08 ± 8.17	93.74 ± 7.28	93.23 ± 7.94	0.7633
Median	96	96	96	
Minimum - Maximum	50 - 100	70 - 99	50 - 100	
**Total**	77	23	100	

SBP, systolic blood pressure; DBP, diastolic blood pressure; RR, respiratory rate; and HR, heart rate.

Trauma indices for the 100 emergency room patients included in the cranial angiotomography study were: 1) Glasgow coma scale score 8.19 ± 3.96, 2) RTS 6.09 ± 1.45, 3) ISS 25.97 ± 16.15, and 3) TRISS 80.14 ± 24.46. Patients without BCVI (Group I) had an average Glasgow coma scale score of 8.14 ± 4.02, and patients with BCVI (Group II) had an average Glasgow coma scale score of 8.35 ± 3.86. Patients in Groups I and II presented with an average RTS of 6.10 ± 1.45 and 6.05 ± 1.45, respectively. Patients in Groups I and II showed an average ISS of 23.13 ± 12.32 and 35.48 ± 22.94, respectively. Patients in Groups I and II presented with an average TRISS of 83.97% ± 21.16% and 67.30% ± 30.34%, respectively. The ISS and TRISS values for Groups I and II were statistically significantly different (Table 3).

**Table 3:** Index of severity in the 100 patients that underwent cervical angiotomography.

	Groups		Total	p-value
	I (without Injury)	II (with injury)		
**GCS**				
Average ± SD	8.14 ± 4.02	8.35 ± 3.86	8.19 ± 3.96	0.6818
Median	7	8	7	
Minimum - Maximum	3 - 15	3 - 15	3 - 15	
Total	77	23	100	
**RTS**				
Average ± SD	6.1 ± 1.45	6.05 ± 1.45	6.09 ± 1.45	0.8205
Median	5,967	6	5,983	
Minimum - Maximum	3 - 8	3,221 - 8	3 - 8	
Total	77	23	100	
**ISS**				
Average ± SD	23.13 ± 12.32	35.48 ± 22.94	25.97 ± 16.15	0.0149*
Median	22	27	22	
Minimum - Maximum	6 - 75	6 - 75	6 - 75	
Total	77	23	100	
**TRISS**				
Average ± SD	83.97 ± 21.16	67.3 ± 30.34	80.14 ± 24.46	0.0235*
Median	94	74	93	
Minimum - Maximum	3 - 100	5 - 99	3 - 100	
Total	77	23	100	

GCS, score on the Glasgow Coma Scale; RTS, revised trauma scale score; ISS, injury severity score; and TRISS, trauma injury severity score, which shows the probability of survival based on the correlation between the revised trauma score, the severity score of the injury, the mechanism of trauma, and the age of the patient. *Indicates a statistically significant difference.

The number of times that the inclusion criteria were present in the total population of 100 patients included: 44 with fractured facial bones (44%), including 14 LeFort II (14%), 18 LeFort III (18%), and 12 simultaneous LeFort II and III (12%); 37 with fractured cervical vertebra (37%); 24 with anisocoria/signs of Horner Syndrome (24%); 13 with a score below eight on the Glasgow coma scale without finding justification on the CT of the skull (13%); 14 with a fracture of the base of the skull 14 (14%); 12 with a nonexpanding cervical hematoma (12%); nine with epistaxis (9%); three with unilateral neurological deficits unexplained after cranial CT scan (3%); four with cerebral infarction identified on tomography (4%); and none showed signs of seatbelt marks above the clavicle (0%).

In the Group I patients, the number of times that the inclusion criteria were present was as follows: 33 with fractured facial bones (42.90%), including 11 LeFort II (14.30%), 14 LeFort III (18.20%), and eight simultaneously LeFort II and III (10.40%); 30 with fracture of the cervical vertebra (39%); 18 with aniscoria/signs of Horner Syndrome (23.40%); 11 with a score lower than eight on the Glasgow coma scale without finding justification on the CT of the skull (14.30%); 12 with fracture of the base of the skull (15.60%); 11 with nonexpanding cervical hematomas (14.30%); six with epistaxis (7.8%); three with unilateral neurological deficit unexplained after cranial CT scan (3.90%); two with cerebral infarction identified on tomography (2.60%); and none showed signs of seatbelt marks above the clavicle, cervical blow, or shock.

In the Group II patients, the number of times that the inclusion criteria were present was follows: 11 with fractured face bones (47.80%), including three LeFort II (13%), four LeFort III (17.40%), and four simultaneously LeFort II and III (17.40%); seven with fracture of the cervical vertebra (30.40%); six with aniscoria/signs of Horner Syndrome (26.10%); two with a score lower than eight on the Glasgow coma scale without finding justification on the CT of the skull (8.70%); two with fracture of the base of the skull (8.70%); one with nonexpanding cervical hematoma (4.30%); three with epistaxis (13%); none with unilateral neurological deficits unexplained after cranial CT scan (0%); two with cerebral infarction identified on tomography (8.70%); none with signs of seatbelt marks above the clavicle, cervical blow, or shock.

The statistical analyses were performed by correlating the inclusion criteria of the total population of 100 patients by comparing Groups I and II. There were no statistically significant differences between Groups I and II.

In the 23 patients of Group II, 12 carotid artery injuries were identified, including: one injury of the common right carotid artery (8.33%); six injuries of the right internal carotid artery (49.93%); and four injuries of the left internal carotid artery (33.33%). Eleven patients had injuries of the vertebral arteries: eight on the left side (72.7%), two of which had concomitant injuries of the subclavian artery, and three on the right side (27.2%).

None of the patients presented with both carotid and vertebral injuries. Four patients showed vascular injuries that extended beyond the topography of the cervical region: one patient had an injury of the meningeal artery; one patient had an injury of the occipital arteries, maxilar and facial; one patient had thrombosis of the right transverse sinus and right sigmoid sinus; and one patient had a pseudoaneurysm of the spinal artery.

The distribution of the 23 patients in Group II with BCVI based on the degree of injury severity included: seven patients with Degree I injuries, ten patients with Degree II injuries, four patients with Degree IV injuries, one patient with a Degree V injury, and one patient with a carotid fistula (Table 4).

**Table 4:** Degree of carotid and vertebral artery injuries in the 23 patients comprising Group II.

Degree of arterial injury	Vertebral arteries	Carotid arteries	Total
Degree I	4	3	7
Degree II	5	5	10
Degree III	-	-	-
Degree IV	2	2	4
Degree V	-	1	1
Thrombosis	-	-	-
Fistula	-	1	1
**Totals**	11	12	23

The treatment of the 23 patients in Group II with BCVI was as follows: 15 patients underwent anticoagulation therapy with heparin (two of the 15 patients also underwent open heart surgery to correct only the subclavian artery injuries), two patients were only observed, and six patients were treated using endovascular methods (one patient underwent collocation of a stent, and five patients underwent gelfoam embolization).

Of the 77 patients in Group I, who did not exhibit BCVI, 14 patients died (18.1%) and 63 patients survived (81.8%). Out of the 63 surviving patients, 16 showed sequelae of trauma (25.3%), and six had other complications (9.52%). The sequelae of the trauma in the 16 Group I patients included: two with paresthesias, two with tetraplegias, five with paresis, and seven with hemiplegias. The complications in the six patients of Group I included: respiratory failure in one patient, hemodynamic instability in one patient, sepsis in one patient, deep vein thrombosis in one patient, acute renal failure in one patient, and multiple organ failure in one patient.

Of the 23 patients in Group II, who presented with BCVI, seven patients died (30.4%) and 16 patients survived (69.5%). Of the 16 surviving patients, 10 had sequelae, including brachial plexus injury in one patient, paresthesia in one patient, tetraplegias in three patients, and paresis in five patients. One patient had complications during the hospitalization, including deep vein thrombosis.

The mortality rate among the 100 patients of the study was 21%. When comparing the mortality rates between Groups I and II, there was no statistically significant difference. A statistically significant difference was observed when comparing TRISS values between the group of 79 patients that survived and the group of 21 patients that died (Table 5). A statistically significant difference was not identified when comparing the actual percentage of survivors in Groups I and II with their respective probabilities of survival calculated by the TRISS score (Table 6).

**Table 5:** Comparison of the probability of survival by TRISS among the patients that survived (79) or died (21).

TRISS	Death		Total	p-value
	No	Yes		
Average ± SD	85.13 ± 19.66	61.38 ± 31.4	80.14 ± 24.46	0.0004*
Median	94	72	93	
Minimum-Maximum	9 - 100	3 - 99	3 - 100	
Total	79	21	100	

*Indicates a statistically significant difference.

**Table 6:** Comparison between the actual percentage of survivors with the predicted percentage of survivors calculated by TRISS.

Group	n	Death (actual)	Survival (actual)	Probability of survival (Average TRISS)	Z	p-value
Without carotid and vertebral artery injuries (Group I)	77	18.18%	81.82%	83.97%	0.34	0.7318
With carotid and vertebral injuries (Group II)	23	30.43%	69.57%	67.30%	0.01	0.9928

## Discussion

It is notable that the large majority of the 100 patients in the current study showed trauma to various body segments with diffuse pain, which is supported by the average ISS of nearly 26 and is characteristic of severely ill people. Furthermore, 44 of the patients had fractures of the facial bones, which is also a source of pain. On the other hand, out of the total of 100 patients, 24 had anisocoria/signs of Horner syndrome; 12 had cervical hematomas; and nine had epistaxis. However, only four presented with cerebral infarction identified in a CT scan of the cranium. Therefore, the pain, signs of bleeding, and signs of Horner syndrome are valuable and should be considered.

Multicenter trials performed in the 1990's identified an incidence of 0.08% and 0.017% of BCVI in specialized trauma care hospitals [2,7-9]. In other studies, the reported BCVI incidence was higher, ranging from 0.24% to 0.50% [3,4]. A recent study reported BCVI incidence rates of up to 1.0% [10]. The authors of this recent study argue that the incidence has increased due to enhanced diagnosis associated with more specific screening in patients with asymptomatic cranial and neck trauma without cerebral ischemia. In the current study, the incidence of BCVI in 100 asymptomatic patients, who were admitted during a 30-month period, was 0.93%.

A retrospective study by Fabian *et al*. spanning 11 years analyzed patients with carotid artery injuries resulting from blunt trauma and identified one or more risk factors that could be used in future studies to identify patients with BCVI in advance of cerebral ischemia [3]. Biffl *et al*.[11] selected asymptomatic patients using seven risk criteria for cervical vessel injury and observed an increase in the incidence of BCVI of between 0.1% to 1.1% over a two and a half year period.

The employment of criteria to identify patients with BCVI should lead to an increased incidence of cervical vessel injury diagnosis. On the other hand, the use of more specific imaging methods that are less invasive or noninvasive, such as angiotomography or angioresonance imaging, will inevitably raise the cost of trauma care. Ideally, the most frequently occurring criteria should be identified and a limited number of criteria for screening should be used to improve the rate of diagnosis without excessive cost increases.

In the current study, 11 inclusion criteria were selected to identify trauma patients with BCVI. These criteria included clinical signs and symptoms and alterations identified in simple radiographs. The overall goal of the current study was to analyze related criteria used in previous studies to determine which criteria were most predictive of BCVI. Unfortunately, we did not identify any criteria that distinguished between the patient groups with and without BCVI.

The current study also examined the number of BCVI criteria met by each patient. Out of the 23 patients with BCVI, there was no significant relationship between the number of BCVI criteria met and BCVI occurrence. It is possible that a future study with a larger patient group would conclude that the use of multiple criteria is not necessary. However, based on the results of the current study, we conclude that all 11 criteria should be used to identify BCVI in blunt trauma patients.

Biffl *et al*. studied problems associated with BCVI over a period of 9 years. One of the objectives of that study was to identify associated or independent risk criteria that could cause BCVI [1,2,6,7]. Through multivariate analysis of the criteria used, they found that a score less than or equal to 6 on the Glasgow coma scale, a petrous bone fracture, diffuse axonal injury, and LeFort II or III type facial fractures correlated significantly with carotid and vertebral artery injuries caused by blunt trauma. Fracture of cervical vertebrae was identified as a unique predictive risk criteria and was independent of vertebral artery injury in blunt trauma. Previous Brazilian studies have not defined BCVI incidence or associated risks. In the current study, we identified a 0.93% incidence of BCVI in a group of 100 blunt trauma patients, but we did not identify any specific risk factor that was more predictive than the others.

A study of risk factors associated with vertebral artery injuries argues that only patients with cervical spine dislocation fractures that extend to the transverse foramen and patients with fractures of the vertebrae from C1 to C3 should be studied in systematic trauma service clinical trials [12].

In the current study, the average Glasgow coma scale score of the 100 patients was nearly 9, and a significant difference was not observed between patients with and without BCVI. The average RTS of the population of 100 patients was approximately 6, and a statistically significant difference was not detected between Groups I and II.

In contrast, the average ISS of the 100 patients was approximately 26, with an average ISS of 23 for patients without BCVI (Group I), and an average ISS of 35.5 for patients with BCVI (Group II). Group II showed a statistically significantly higher ISS average, indicating greater severity. A similar result was seen with regard to the probability of survival as observed using TRISS. The probability of survival was significantly lower among Group II patients (67%) than among Group I patients (84%), and the average probability of survival among all 100 patients was 80% (Table 5). Mortality among all 100 patients was 21%, mortality for Group I was 18%, and mortality for Group II was 30.5%. A comparison between the actual survival percentage and the predicted survival percentage calculated by TRISS showed that they were not statistically significantly different (Table 6).

There are several aspects of angiotomography that make it very useful tool for studying BCVI, especially in asymptomatic patients. A key aspect of angiotomography is that the vast majority of patients for which cervical artery study is indicated also require tomography to investigate other injuries. As a result, the patient does not require an additional referral to study the cervical vessels. Angiotomography of the carotid and vertebral arteries would then be analyzed together with other injuries, such as cerebral injury, fractures of the face or base of the skull, and injuries of other cervical region structures, such as the vertebral column.

In the current study, all 100 patients underwent cervical angiotomography and no abnormalities were identified in the images, demonstrating a high degree of confidence in the resolution. Carotid and vertebral artery injuries were identified in 23 patients (23%). Of the 23 BCVI patients that underwent angiotomography, six (26%) underwent angiography for therapeutic procedures (five embolizations and one collocation of a stent). One patient out of the 77 that did not show BCVI suffered acute renal failure, caused by the use of contrast, but recovered without permanent sequelae.

The reported occurrence of carotid and vertebral artery injury with blunt trauma is highly variable among published studies. The major confounding factor is that the vast majority of cases show separate studies of the carotid and vertebral arteries. Few studies have reported the simultaneous investigation of all four arteries. One such study by Miller *et al*. performed angiography in 216 patients with risk factors for cervical artery injuries over a period of two years; they identified 24 patients with carotid artery injuries (11.11.%) and 43 patients with vertebral artery injuries (19.71%) [10]. Another study carried out by McKinney *et al*. performed angiography on 71 patients with risk factors for cervical artery injuries over the course of 13 months; they identified 12 patients with carotid artery injuries (16.67%) and 12 patients with vertebral artery injuries (16.67%) [13].

In the current study, 12 out of the 100 patients with risk factors for carotid and vertebral artery injuries were diagnosed with vertebral injuries. The results of the current study are very similar to those of McKinney *et al*., with only a slightly smaller incidence of carotid and vertebral injury in the current study. McKinney *et al*. studied 24 patients with carotid and vertebral injuries and identified 10 patients with Degree I injuries, four patients with Degree II injuries, eight patients with Degree III injuries, two patients with Degree IV injuries, and no patients with Degree V injuries [13]. In the current study we identified seven patients with Degree I injuries, ten patients with Degree II injuries, no patients with Degree III injuries, four patients with Degree IV injuries, one patient with Degree V injuries, and one patient with a fistula.

Fabian *et al*. studied 67 patients with 87 carotid injuries, including 54 dissections, 11 pseudoaneurysms with dissections, 17 thromboses, four carotid-cavernous fistulas, and one transection. The patients were treated in the following manner: the fistulas were embolized with a balloon, the transection was clamped, 47 of the patients were treated with heparin, eight patients were only observed, six patients received aspirin, and one patient was submitted for surgery. In that study, the group of patients that received heparin showed greater improvement than those who did not receive heparin. The complications that occurred in patients who received heparin included: gastrointestinal hemorrhage, hemorrhage of the hepatic artery, tracheal hemorrhage, two subdural hematomas that required surgery, and worsening of a ventricular hemorrhage. Subsequently, when 39 patients were reexamined, 62% showed normalization of the injury and 29% had developed a pseudoaneurysm [3].

Biffl *et al*. identified 114 patients with 157 injuries of the carotid arteries, and 79 patients with 97 vertebral artery injuries. In that study, 137 were Degree I injuries; 52 were Degree II injuries; 32 were Degree III injuries, 25 were Degree IV injuries; and eight were Degree V injuries. One week after trauma, 114 carotid injuries and 65 vertebral injuries were reevaluated with angiography, and 82% of the Degree IV injuries and 93% of the Degree III injuries showed no change. In contrast, 57% of the Degree I injuries and 8% of the Degree II injuries regressed to complete normality and treatment was discontinued. However, 8% of the Degree I injuries and 43% of the Degree II injuries worsened and developed pseudoaneurysms, requiring interventional treatment. Biffl *et al*. concluded that follow-up angiography can change the treatment in up to 61% of Degree I and II injuries [14].

In 2005, Cothren *et al*. published a prospective study and verified that patients who presented with a carotid pseudoaneurysm and were treated with a stent represented 21% of complications by occlusion of up to 45%. On the contrary, patients who were treated with an antithrombotic agent represented 5% of arterial occlusions. None of the asymptomatic patients had arterial obstructions with antithrombotic agents. Cothren *et al*. concluded that treatment with antithrombotic agents remains the best therapeutic option and that the use of stents remains controversial [15]. In 2008, Berne *et al*. defended the use of stents in the carotid artery as being a safe and effective initial therapy for patients with pseudoaneurysms without carotid obstruction. The incidence of morbidity up to four years was very small [16].

The ability to treat patients with improved neurological results is the desire of all trauma teams, however clinical complexities are associated with every patient. In the current study, 15 patients underwent treatment with heparin: five patients were treated with non-fractionated heparin, and 10 patients were treated with fractionated heparin. Of the three patients that died, two were the result of brain death. Four out of the eight patients not treated with heparin died, and two were due to brain death. Two patients were observed clinically, and six patients underwent endovascular treatment. In summary, 17 patients were treated clinically and six patients were treated using endovascular methods. No complications occurred in patients treated clinically with heparin or in patients who underwent endovascular treatment. Taken together, the results of the current study suggest that treatment decisions should be made based on the experience of the clinicians and on the clinical and neurological status of the patient.

In summary, the results of this study indicate that:

1. The incidence of carotid and vertebral artery injuries in blunt trauma was 0.93%.

2. Patients with carotid and vertebral artery injuries showed higher severity indices than those without carotid and vertebral injuries, but showed similar mortality rates.

3. Based on the eleven primary criteria analyzed in the current study, a clear set of criteria for the indication of angiotomography remains to be established.

## Conclusions

Although there is no consensus regarding the criteria that should be used to indicate angiotomography for BCVI diagnosis in blunt trauma patients, we conclude that the criteria used in the current study led to a diagnosis of BCVI in 0.93% of 2,467 trauma patients, BCVI injuries were associated with more severe traumas and did not affect mortality.

## Competing interests

The authors declare that they have no competing interests.

## Authors' contributions

PR participated in the study of the angiotomography. PSR carried out the BCVI studies, participated in the sequence alignment and drafted the manuscript. FB participated in the sequence alignment, analysis and interpretation of datas. JA participated in the design of the study and performed the statistical analysis. GG participated in the study of the imagem and award of the angiotomography. BD participated in the coordination and study of blunt trauma. All authors read and approved the final manuscript.

## References

[B1] Biffl WL Moore EE Offner PJ Burch JM Blunt carotid and vertebral arterial injuries World J Surg 2001 25 1036 1043 10.1007/s00268-001-0056-x 11571969

[B2] Miller PR Fabian TC Bee TK Timmons S Chamsuddin A Finkle R Croce MA Blunt cerebrovascular injuries diagnosis and treatment J Trauma 2001 51 2 279 286 10.1097/00005373-200108000-00009 11493785

[B3] Fabian TC Patton JH Jr Croce MA Minardd G Kudsk KA Pritchard FE Blunt carotid injury. importance of early diagnosis and anticoagulant therapy Ann Surg 1996 223 513 10.1097/00000658-199605000-00007 8651742 PMC1235173

[B4] Punjabi AP Plaisier BR Haug RH Malangoni MA Diagnosis and management of blunt carotid artery injury in oral and maxillofacial surgery J Oral Maxillofac Surg 1997 55 1388 10.1016/S0278-2391(97)90634-0 9393397

[B5] Ramadan F Rutledge R Oller D Howell P Baker C Keagy B Hill C Carotid artery trauma: a review of contemporary trauma center experiences J Vasc Surg 1995 21 46 10.1016/S0741-5214(95)70243-1 7823361

[B6] Biffl WL Moore EE Elliott JP Brega KE Burch JM Blunt cerebrovascular injuries Curr Prob Surg 1999 36 507 10.1016/s0011-3840(99)80807-7 10394346

[B7] Biffl WL Egglin T Benedetto B Gibbs F Cioffi WG Sixteen-slice computed tomographic angiography is a reliable noninvasive screening test for clinically significant blunt cerebrovascular injuries J Trauma 2006 60 4 745 51 10.1097/01.ta.0000204034.94034.c4 16612293

[B8] Biffl WL Diagnosis of blunt cerebrovascular injuries Curr Open Critic Care 2003 9 6 530 4 10.1097/00075198-200312000-00011 14639074

[B9] Martin RF Eldrup-Jorgensen J Clark DE Bredenberg CE Blunt trauma to the carotid arteries J Vasc Surg 1991 14 789 10.1067/mva.1991.32076 1960809

[B10] Miller PR Fabian TC Croce MA Cagiannos C Williams JS Vang M Qaisi WG Felker RE Timmons SD Prospective screening for blunt cerebrovascular injuries: analysis of diagnostic modalities and outcomes Ann Surg 2002 236 386 395 10.1097/00000658-200209000-00015 12192325 PMC1422592

[B11] Biffl WL Moore EE Offtner PJ Brega KE Franciose RJ Burch JM Blunt carotid arterial injurries: implications of a new grading scale J Trauma 1999 47 5 845 10.1097/00005373-199911000-00004 10568710

[B12] Cothren CC Moore EE Biffl WL Ciesia DJ Ray CE Jr Johnson JL Moore JB Burch JM Cervical spine fracture patterns predictive of blunt vertebral artery injury J Trauma 2003 55 5 811 3 10.1097/01.TA.0000092700.92587.32 14608149

[B13] McKinney A Ott F Short J McKinney Z Truwit C Angiographic frequency of blunt cerebrovascular injury in patients with carotid canal of vertebral foramen fractures on multidetector CT Eur J Radiol 2007 62 3 385 93 10.1016/j.ejrad.2007.01.008 17399930

[B14] Biffl WL Ray CE Jr Moore EE Franciose RJ Somer Aly S Heyrosa MG Johnson JL Burch JM Treatment-related outcomes from blunt cerebrovascular injuries - importance of routine follow-up arteriography Ann Surg 2002 235 5 699 707 10.1097/00000658-200205000-00012 11981216 PMC1422496

[B15] Cothren CC Moore EE Ray CE Jr Ciesla DJ Johnson JL Moore JB Burch JM Carotid artery stents for blunt cerebrovascular injury - risks exceed benefits Arch Surg 2005 140 480 486 10.1001/archsurg.140.5.480 15897444

[B16] Berne JD Reuland KR Villareal DH McGovern TM Rowe SA Norwood SH Internal carotid artery stending for blunt carotid artery injuries with an associated pseudoaneurysm J Trauma 2008 64 2 398 405 10.1097/TA.0b013e31815eb788 18301205

